# Age and Sex Invariance of the Woodcock-Johnson IV Tests of Cognitive Abilities: Evidence from Psychometric Network Modeling

**DOI:** 10.3390/jintelligence9030035

**Published:** 2021-07-07

**Authors:** Okan Bulut, Damien C. Cormier, Alexandra M. Aquilina, Hatice C. Bulut

**Affiliations:** 1 Centre for Research in Applied Measurement and Evaluation, University of Alberta, Edmonton, AB T6G 2G5, Canada; damien.cormier@ualberta.ca; 2 Department of Educational Psychology, University of Alberta, Edmonton, AB T6G 2G5, Canada; aquilina@ualberta.ca; 3 Department of Educational Sciences, Cukurova University, Adana 01250, Turkey; hcyavuz@cu.edu.tr

**Keywords:** intelligence, cognitive abilities, invariance, sex, age, psychometric network modeling

## Abstract

The Woodcock-Johnson IV Tests of Cognitive Abilities (WJ IV COG) is a comprehensive assessment battery designed to assess broad and narrow cognitive abilities, as defined by the Cattell-Horn-Carroll (CHC) theory of intelligence. Previous studies examined the invariance of the WJ assessments across sex and age groups using factor analytic methods. Psychometric network modeling is an alternative methodology that can address both direct and indirect relationships among the observed variables. In this study, we employed psychometric network modeling to examine the invariance of the WJ IV COG across sex and age groups. Using a normative sample (*n* = 4212 participants) representative of the United States population, we tested the extent to which the factorial structure of the WJ IV COG aligned with CHC theory for the school-aged sample. Next, we used psychometric network modeling as a data-driven method to investigate whether the network structure of the WJ IV COG remains similar across different sex and age (age 6 to 19, inclusively) groups. Our results showed that the WJ IV COG maintained the same network structure across all age and sex groups, although the network structure at younger ages indicated weaker relationships among some subtests. Overall, the results provide construct validity evidence for the WJ IV COG, based on both theoretical and data-driven methods.

## 1. Introduction

Researchers and practitioners often use a standardized measure to assess an individual’s general cognitive functioning, as well as their specific cognitive abilities. The Woodcock-Johnson IV Tests of Cognitive Abilities (WJ IV COG; Schrank et al. 2014) is an example of a standardized measure that is used in educational, clinical, and research settings. The WJ IV COG is a comprehensive assessment battery designed to measure various cognitive abilities, as defined by the Cattell-Horn-Carroll (CHC) theory of intelligence. Compared with its predecessor, the WJ III COG (Woodcock et al. [2001] 2007), the WJ IV COG provides a test battery of intelligence that is more aligned with recent research on CHC theory, while preserving the psychometric qualities (e.g., reliability and validity) from previous versions (Reynolds and Niileksela 2015).

The authors of the WJ IV COG provide evidence for the factor structure of the entire battery across different age groups (Schrank et al. 2014). Additionally, the technical report for the WJ IV COG presents the results of differential item functioning analyses across sex, race, and ethnicity, indicating that the problematic items have been eliminated from the test. However, the authors did not examine the invariance of the battery across sex and age groups. A very common method for evaluating the invariance of psychological measures, such as the WJ IV COG, across various groups is measurement invariance testing based on multi-group confirmatory factor analysis (CFA; Milfont and Fischer 2010). This procedure enables researchers to fit the same CFA model across multiple groups and examine the configural, metric, scalar, and strict invariance of the factorial structure. However, the measurement invariance approach is known to be highly sensitive to sample size, leading to the rejection of measurement invariance due to small disparities among the groups (Putnick and Bornstein 2016; van Dijk et al. 2021).

In the current study, we perform psychometric network analysis using the United States normative data of the WJ IV COG and examine the invariance of the WJ IV COG network structure based on sex and age groups. First, we apply a hierarchical CFA model to verify the factorial structure of the WJ IV COG based on CHC theory. Next, we perform psychometric network modeling to delineate the network structure of the CHC cognitive abilities measured within the WJ IV COG. Finally, we use the network model tree (NMT; Jones et al. 2020) approach to evaluate the impact of sex and age on the network structure of the WJ IV COG. The NMT approach recursively splits the data based on covariates (i.e., sex and age) to detect significant differences in the network structure. As we discuss the implications of our findings for practitioners using the WJ IV COG, we also show how the NMT approach can guide researchers when testing network invariance with psychological measures.

## 2. Literature Review

### 2.1. CHC Theory

Many of the well-known and contemporary intelligence test batteries, including the WJ IV COG, define and measure different aspects of intelligence based on CHC theory (Keith and Reynolds 2010). Therefore, a brief review of CHC theory and its components seems relevant to understanding the assessment of intelligence within and across various measures of intelligence. CHC theory was created by merging aspects of Spearman’s (1904) g, the Horn–Cattell Gf–Gc theory (Horn and Cattell 1966a; Horn and Noll 1997), Thurstone’s (1938) broad cognitive abilities, and Carroll’s (1993) three-stratum theory (Niileksela and Reynolds 2014). As a multi-factorial and hierarchical structure of intelligence, CHC theory consists of more than 70 narrow abilities at the first level (i.e., stratum); approximately 10 broad abilities at the second level; and general intelligence, or g, at the third level (Kaufman et al. 2012; Niileksela and Reynolds 2014). A comprehensive review of the CHC framework and its role in the investigation of the structure of human intelligence can be found in McGrew’s (2009) editorial. 

Although there are ten broad abilities identified within CHC theory, seven of these broad abilities are more commonly measured: comprehension-knowledge (Gc), fluid reasoning (Gf), short-term memory (Gsm), processing speed (Gs), auditory processing (Ga), visual-spatial ability (Gv), and long-term storage and retrieval (Glr). Comprehension-knowledge (Gc) is the ability to use previous experience, knowledge, and skills, which are valued by one’s culture, to communicate or reason in unique situations. Fluid reasoning (Gf) is defined as the ability to control one’s attention to solve novel problems, without the ability to rely on previous knowledge or schemas. Short-term memory (Gsm) is the ability to encode, maintain, and manipulate information while it is in one’s immediate consciousness. Processing speed (Gs) is the ability to execute simple and repetitive cognitive tasks rapidly and effortlessly. Auditory processing (Ga) is the ability to identify and process meaningful, nonverbal information in sound. Visual processing (Gv) is the ability to use simulated mental imagery to solve problems, and long-term storage and retrieval (Glr) is the ability to store, solidify, and then retrieve information over time (see Schneider and McGrew (2012) for a more comprehensive explanation of CHC broad abilities).

Several well-known tests of intelligence, such as the WJ IV COG, KABC-II, and WISC-V, are aligned closely with the hierarchical structure of the general and broad cognitive abilities from CHC theory. More specifically, the WJ IV COG consists of 18 subtests that measure general intellectual ability, as well as broad and narrow cognitive abilities based on CHC theory (Schrank et al. 2014). The Standard Battery of the WJ IV COG can be used to compute scores for three general intelligence composites: the General Intellectual Ability (GIA) based on the Gc, Gf, Gwm, Gs, Ga, Glr, and Gv broad abilities; the Brief Intellectual Ability (BIA) based on the Gc, Gf, and Gwm broad abilities; and an additional composite consisting of only Gf and Gc. The WJ IV COG also offers scores for the CHC narrow abilities (perceptual speed, quantitative reasoning, number facility, and cognitive efficiency) and a clinical cluster score. The psychometric properties of the WJ IV COG standard and extended subtests can be found in the Technical Manual (Schrank et al. 2014). Additionally, Reynolds and Niileksela (2015) provide a technical review of the WJ IV COG for both researchers and practitioners.

### 2.2. Sex and Age Differences in Intelligence

To date, several studies have examined sex differences in the general intellectual ability and broad abilities underlying well-known intelligence tests, such as the WJ III COG (e.g., Keith et al. 2008), the Kaufman Assessment Battery for Children—Second Edition (KABC-II; e.g., Hajovsky et al. 2018; Reynolds et al. 2008), the Wechsler Intelligence Scale for Children—Fifth Edition (WISC-V; e.g., Chen et al. 2015; Chen et al. 2020; Dombrowski et al. 2020), and the Wechsler Adult Intelligence Scale—Fourth Edition (e.g., Pezzuti et al. 2020). Generally, research on sex differences in general intelligence (g) has been inconsistent. Most studies have reported that males tend to outperform females (e.g., Flores-Mendoza et al. 2013; Jackson and Rushton 2006; Lynn and Irwing 2004), whereas others have concluded that females score higher on the g factor (e.g., Arden and Plomin 2006; Reynolds et al. 2008). 

Inconsistencies regarding sex differences also occur in the context of broad cognitive abilities. Most studies have reported that females may have an advantage in processing speed (Gs). Using the earlier forms of the WJ assessments, Camarata and Woodcock (2006) found that females scored significantly higher on the tests of Gs, with the largest difference occuring in adolescent subgroups. Similar findings on female superiority in Gs have been reported by other researchers (e.g., Burns and Nettelbeck 2005; Keith et al. 2008; van der Sluis et al. 2006). Other studies reported that males outperform females on the broad cognitive abilities of working memory (Gwm), visual-spatial ability (Gv), and crystallized intelligence (Gc) (Dolan et al. 2006; Keith et al. 2008; Reynolds et al. 2008). More recent studies using the WISC-V reported no sex differences based on investigation of the factorial invariance of the subtests (e.g., Chen et al. 2015, 2020). 

Numerous studies have reported that sex differences in intelligence vary between age groups and over time. Lynn’s (1994, 1999) developmental theory of sex differences in intelligence states that there is an intellectual difference between males and females due to the differing rates at which each sex matures. The progression of maturity accelerates for females when they are around nine years of age and remains ahead of males until the age of 14 or 15 (Colom and Lynn 2004). After that point, females begin to decelerate compared with males, and males continue to mature and grow. Differential rates of maturation between females and males are also expected to be influential on the growth of their intelligence. For example, Colom and Lynn (2004) found that females performed better at younger ages compared with males, but among the older age groups, the performance among females declined in relation to males. Lynn et al. (2004) reported similar findings in a study where they examined sex differences in fluid intelligence and g using a sample of 12- to 18-year-old participants. Although there are further studies indicating a significant interaction between age and sex in intelligence (e.g., Arden and Plomin 2006; Lynn and Kanazawa 2011), other researchers reported findings that were inconsistent with Lynn’s developmental theory (e.g., Keith et al. 2008; Savage-McGlynn 2012).

Several studies have found that there are differences between females and males at various ages but that generally there is not a systematic pattern to these differences. For example, Keith et al. (2008) explored sex differences in participants aged six to fifty-nine in the general and broad cognitive abilities underlying the WJ III COG. The authors reported that females and males showed a consistent advantage in processing speed (Gs) and comprehension–knowledge (Gc), respectively. However, there were no significant sex differences in auditory processing (Ga), short-term memory (Gwm), long-term retrieval (Glr), and fluid reasoning (Gf). In contrast to the developmental theory suggesting a male advantage during adulthood, significant and consistent sex differences were reported in general ability, favoring females during both adolescence and adulthood. Keith et al. (2008) reported that females, at all ages, demonstrated an advantage in processing speed (Gs) and short-term memory (Gwm), while males showed an advantage in visual-spatial ability (Gv) from ages eight and older. Some studies also reported a consistent measurement of general and broad cognitive abilities across all ages (e.g., Reynolds et al. 2007).

### 2.3. Methodological Considerations

To date, most studies have employed latent variable modeling for studying sex and age differences in the cognitive abilities underlying performance in tests of intelligence (e.g., Chen et al. 2015; Dombrowski et al. 2020; Reynolds et al. 2007, 2008; Taub and McGrew 2004). Modeling such differences within a latent variable framework allows researchers to test for factorial invariance across groups and detect significant differences in latent mean levels and the variability of cognitive abilities. When testing sex and age differences with latent variable modeling, the most widely used approach is multi-group CFA. Generally, researchers use a multi-group CFA approach for testing measurement invariance of the factorial structure underlying intelligence tests. This procedure involves the comparison of hierarchically nested, multi-group CFA models for testing configural, metric, scalar, and strict invariance (see Putnick and Bornstein (2016) for a detailed review of measurement invariance testing and reporting). 

Despite its utility in evaluating the construct equivalence of intelligence tests across demographic groups and across time, the measurement invariance approach has some limitations in practice. First, the sample size may moderate the sensitivity of measurement invariance. Model comparison based on the change in chi-square (χ^2^) for two hierarchically nested models is known to be highly sensitive to sample size and thereby lead to measurement non-invariance in large samples due to small differences among the groups (French and Finch 2006; Putnick and Bornstein 2016). Second, a similar concern pertains to the number of groups to be compared when testing measurement invariance. As the number of groups compared in tests of measurement invariance increases, the χ^2^ difference test, model fit indices, and associated evaluation criteria may require adjustments to detect the group differences accurately (Rutkowski and Svetina 2014). Thus, researchers often examine sex differences in general and broad cognitive abilities by running tests of measurement invariance across different age groups separately, instead of running simultaneous tests of measurement invariance for sex and age groups. Third, if full measurement invariance is not supported, researchers are forced to look for partial invariance in the model by releasing constraints on factor loadings or intercepts or both. Although there are clear guidelines on establishing partial measurement invariance (e.g., Putnick and Bornstein 2016; van de Schoot et al. 2012), the theoretical consequences of partial invariance for the interpretation of group or developmental differences in cognitive abilities are still not well understood. 

In the present study, we employed psychometric network modeling to explore the stability of dynamic coupling between cognitive abilities across sex and age groups (van der Maas et al. 2017). Although psychometric network modeling (Epskamp et al. 2012) is often considered an exploratory tool to determine the number of factors (or clusters) based on full or partial correlations, it can also be used as a confirmatory tool for the comparison of networks and the cross-validation of networks (Kan et al. 2019). The current paper aimed to evaluate the stability of the network of cognitive abilities extracted from the WJ IV COG across sex and age groups. Using the United States normative sample of children and adolescents ranging from 6 to 19 years in age, we examined whether sex and age would lead to significant differences in the network structure of the WJ IV COG. A new psychometric network approach—the NMT approach—was used for evaluating network invariance.

## 3. Materials and Methods

### 3.1. Participants

The sample of this study consisted of 4212 participants (aged 6 to 19 years old, *M* = 12.3, *SD* = 4) who participated in the norming study of the WJ IV COG in the United States. The sample is representative of the United States population in terms of individual (e.g., sex, race, parent education) and community variables (e.g., census region and community type). Table 1 presents details of the sample demographics. In this study, the sample was split into four age groups based on the age categorization in the WJ IV technical manual (McGrew et al. 2014): Group 6–8 (*n* = 954), Group 9–11 (*n* = 949), Group 12–14 (*n* = 923), and Group 15–19 (*n* = 1386).

### 3.2. Measures

In this study, we selected 14 subtests from the WJ IV COG to define the following cognitive abilities based on CHC theory: (1) Comprehension-Knowledge (Gc) from the Oral Vocabulary and General Information subtests, (2) Fluid Reasoning (Gf) from the Number Series and Concept Formation subtests, (3) Short-Term Working Memory (Gwm) from the Verbal Attention and Numbers Reversed subtests, (4) Cognitive Processing Speed (Gs) from the Letter-Pattern Matching and Pair Cancellation subtests, (5) Auditory Processing (Ga) from the Phonological Processing and Nonword Repetition subtests, (6) Long-Term Retrieval (Glr) from the Story Recall and Visual-Auditory Learning subtests, and (7) Visual Processing (Gv) from the Visualization and Picture Recognition subtests. Information about these subtests can be found in the WJ IV Technical Manual (Schrank et al. 2014). Scale scores from the WJ IV COG subtests were used in data analysis. The WJ IV COG scale scores follow the W scale, which is based on a direct transformation of the Rasch logit scale with a center of 500 points. 

### 3.3. Data Analysis

#### 3.3.1. Factor Analysis

Confirmatory factor analysis was performed to assess the model fit of the WJ IV COG, assuming a hierarchical factor model based on CHC theory. This hierarchical CFA model consisted of a higher-order latent variable representing general intellectual ability (g), which was defined from seven broad cognitive abilities (Gc, Gf, Gwm, Gs, Ga, Glr, and Gv). Additionally, the seven latent variables representing the broad cognitive abilities were defined from pairs of subtests, for a total of fourteen subtests at this lower level. The hierarchical CFA model was estimated for the entire sample and for the sex (female and male) and age (6–8, 9–11, 12–14, and 15–19) groups separately. Model estimation was completed using the lavaan package (Rosseel 2012) in R (R Core Team 2021). Maximum likelihood with robust standard errors (known as MLR) was used for the model estimation. Hu and Bentler’s (1999) guidelines (i.e., comparative fit index [CFI] and Tucker-Lewis index [TLI] ≥ 0.95; root mean square error of approximation [RMSEA] ≤ 0.06; standardized root mean squared residual [SRMR] ≤ 0.08) were used for evaluating model fit. Additionally, Akaike Information Criteria (AIC) and Bayesian Information Criteria (BIC) values were included to compare the CFA models with the psychometric network models. Smaller AIC and BIC values indicate better fit.

#### 3.3.2. Psychometric Network Analysis

Following the hierarchical CFA model, we used psychometric network modeling (Borsboom 2008; Borsboom and Cramer 2013) to examine the network structure of the W scores derived from the WJ IV COG subtests. The W scale in the WJ IV COG is a direct transformation of the Rasch logit scale (i.e., W = 9.1024 × logits + 500). Psychometric network modeling is used for forming a network structure of observed variables (e.g., items, scores, or symptoms), connected with edges (i.e., correlations, causal relations). In psychological networks, psychological variables are represented by nodes. Edge thickness shows the strength of the relationship between the nodes (e.g., thicker edges indicate stronger relationships). In this study, we conducted psychometric network analyses in two stages. In the first stage, we estimated a Gaussian Graphical Model (GGM; Lauritzen 1996) using the graphical least absolute shrinkage and selector operator (gLASSO) regularization method, based on the partial correlation matrix of the subtest scores from the WJ IV COG. The bootnet (Epskamp et al. 2018) and qgraph (Epskamp et al. 2012) packages in R (R Core Team 2021) were used for estimating the GGM. This overall model helped us examine the network structure of the WJ IV COG, without considering the effects of sex and age. To assess the importance of nodes in the network structure, we computed several centrality indices. These indices quantify how strongly a node is connected to other nodes (node strength), how strongly a node is indirectly connected to other nodes (closeness), and how important a node is in the average pathway between other pairs of nodes (betweenness; Epskamp et al. 2018; Hevey 2018).

In the second stage, we used the NMT approach to examine the invariance of the network structure of the WJ IV COG across sex and age groups. Following the same approach as structural equation model trees (Brandmaier et al. 2013), the NMT approach combines psychometric network modeling with recursive partitioning techniques to detect significant differences in the network structure based on covariates. That is, the NMT approach assesses how covariates are associated with heterogeneity across the network structure (Jones et al. 2020). In this study, we used model-based recursive partitioning (MOB; Zeileis et al. 2008) to split the network structure of the WJ IV COG subtests based on sex and age groups. The MOB algorithm splits the network structure in a way that the network parameters are maximally heterogeneous across the terminal (i.e., leaf) nodes in the tree model. If sex and age groups are associated with statistically significant differences in the network structure, then the MOB algorithm will split the network structure at least once, or more, based on these covariates and create terminal nodes. We estimated network model trees using the MOB algorithm in the networktree package (Jones et al. 2020). For the psychometric network analyses, we followed the guidelines of Epskamp et al. (2018) and Jones et al. (2020). A sample data set and the R codes used in this study can be found at https://osf.io/m7846/ (Bulut 2021).

## 4. Results

### 4.1. Confirmatory Factor Analysis of the WJ IV COG

Model fit indices for the confirmatory factor analyses are presented in Table 2. The overall model refers to the hierarchical CFA model based on the entire sample where the model parameters were constrained to be equal for all groups. Although the overall model appeared to fit the data well, it did not follow some of the model fit guidelines (e.g., RMSEA ≤ 0.06) suggested by Hu and Bentler (1999). Table 2 also shows the model fit indices for the hierarchical CFA models estimated for each sex and age group. These models estimated the variances and covariances among the observed indicators for each sex and age group separately, without any constraints. As shown in the table, the model fit indices for the male and female samples were similar to those from the overall model, whereas the model fit indices for the age groups were relatively worse than the fit values obtained from the overall model. The fit indices shown in Table 2 suggest that the hierarchical CFA model based on CHC theory may not be entirely plausible for some age groups. Although not shown in the table, all first- and higher-order factor loadings were reasonable for the estimated models, supporting the grouping of WJ subtests in defining broad cognitive abilities. Overall, the results of the hierarchical CFA models suggest that although sex may not have a significant impact on the factorial structure of the WJ IV COG, age appears to influence the relationships among the broad cognitive abilities and higher-order latent variables representing the general intellectual ability. 

### 4.2. Psychometric Network Analyses of the WJ IV COG

**Figure 1:**
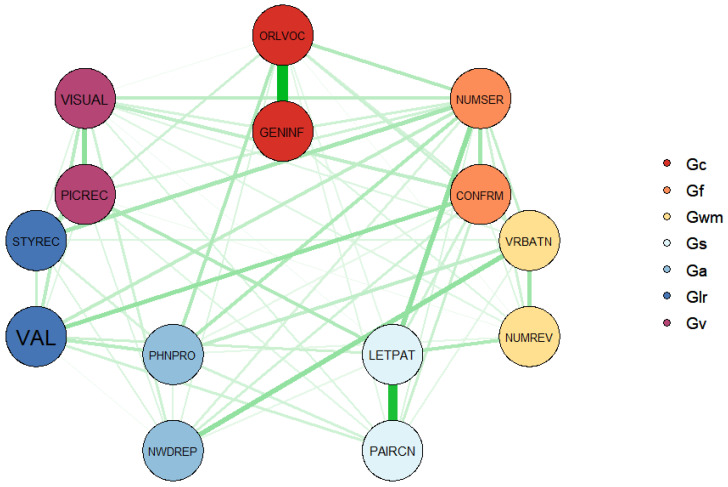
Weighted, undirected network structure of WJ IV COG (Note: ORLVOC: Oral Vocabulary; NUMSER: Number Series; VRBATN: Verbal Attention; LETPAT: Letter-Pattern Matching; PHNPRO: Phonological Processing; STYREC: Story Recall; VISUAL: Visual-Auditory Learning; GENINF: General Information; CONFRM: Concept Formation; NUMREV: Numbers Reversed; NWDREP: Nonword Repetition; VAL: Visualization; PICREC: Picture Recognition; PAIRCN: Pair Cancellation).

The overall psychometric network model demonstrated stronger model fit across all model fit indices, except for the chi-square test; χ^2^(26) = 99.86, *p* < 0.001; CFI = 1.00, TLI = 0.99, RMSEA = 0.026; AIC = 470,272; BIC = 470,861). In particular, smaller AIC and BIC values suggest that the psychometric network model fit the WJ IV COG data better than the hierarchical CFA model did. A graphical representation of the network structure of WJ IV COG is given in Figure 1.

**Figure 2:**
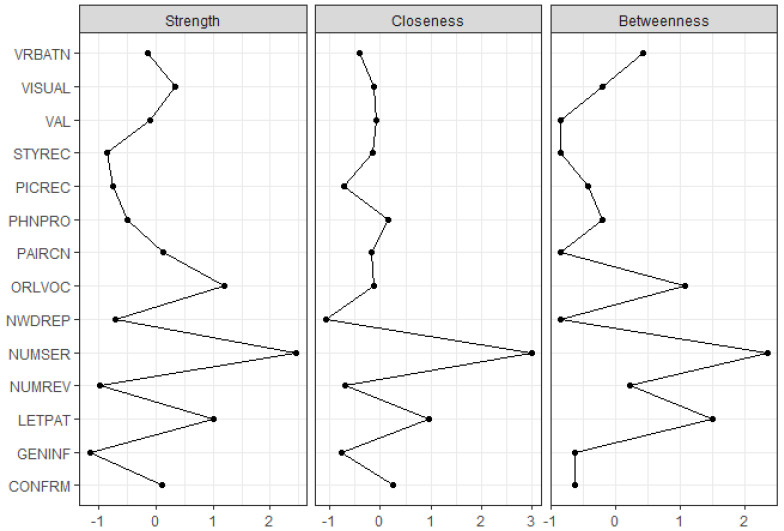
Centrality indices of the nodes in the WJ IV COG network structure (Note: ORLVOC: Oral Vocabulary; NUMSER: Number Series; VRBATN: Verbal Attention; LETPAT: Letter-Pattern Matching; PHNPRO: Phonological Processing; STYREC: Story Recall; VISUAL: Visual-Auditory Learning; GENINF: General Information; CONFRM: Concept Formation; NUMREV: Numbers Reversed; NWDREP: Nonword Repetition; VAL: Visualization; PICREC: Picture Recognition; PAIRCN: Pair Cancellation).

In Figure 1, the color of each node indicates which broad cognitive abilities are defined by the WJ IV COG subtests, while the width (i.e., thickness) and color density of each line (i.e., edge) connecting the nodes represents the strength of association between different pairs of nodes. The two subtest pairs representing the comprehension-knowledge (Gc) and cognitive processing speed (Gs) broad abilities have strong associations within these pairs of subtests, whereas the subtests for the remaining broad abilities appear to be inter-correlated. Of note, the Glr and Ga pairings do not appear to have a strong association to each other. It should be noted that graphical spacing between the nodes does not necessarily indicate the magnitude of the relationship between the WJ IV COG subtests or which subtests are more important than the others (Jones et al. 2018). Therefore, we demonstrate centrality indices for the estimated network structure in Figure 2 to interpret network structure more accurately. The x-axis of Figure 2 indicates standardized z scores in the indices for strength, closeness, and betweenness across the fourteen subtests of the WJ IV COG, with higher values indicating that nodes are more important to the network structure. 

The strength index (on the left-hand side of Figure 2) indicates how strongly each node is connected to the other nodes. Strength values in this study reveal that the Number Series subtest is the most important node for the network structure of the WJ IV COG, followed by Oral Vocabulary and Letter-Pattern Matching. The closeness index (in the middle of Figure 2) indicates each node’s relationship to all other nodes in the network based on the sum of indirect connections from that node (Hevey 2018). Closeness values obtained from the WJ IV COG network model indicate that the Number Series subtest plays a central role in the network, and thus scores from the Number Series subtest can affect the other nodes significantly. Lastly, the betweenness index (on the right-hand side of Figure 2) indicates how important a particular node is in the average pathway between other pairs of nodes (Hevey 2018). In the WJ IV COG network structure, Number Series, followed by Letter-Pattern Matching and Oral Vocabulary, have high betweenness indices. These subtests serve as a gatekeeper (or a bridge) between the other nodes in the WJ IV COG network structure.

**Figure 3:**
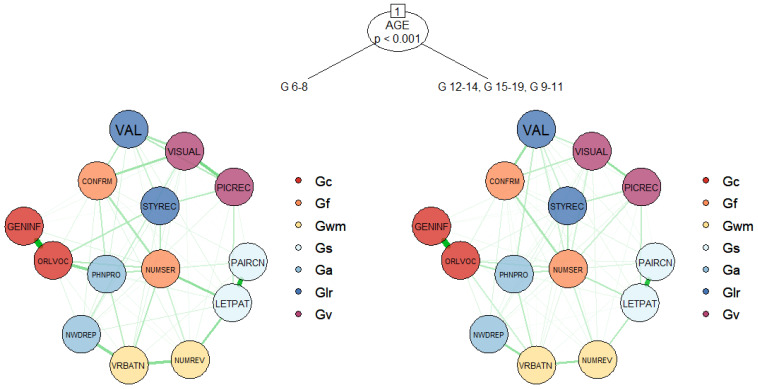
Network model tree of the WJ IV COG based on age and sex (Note. Sex was not included in the figure because it did not lead to any significant differences in the network structure. ORLVOC: Oral Vocabulary; NUMSER: Number Series; VRBATN: Verbal Attention; LETPAT: Letter-Pattern Matching; PHNPRO: Phonological Processing; STYREC: Story Recall; VISUAL: Visual-Auditory Learning; GENINF: General Information; CONFRM: Concept Formation; NUMREV: Numbers Reversed; NWDREP: Nonword Repetition; VAL: Visualization; PICREC: Picture Recognition; PAIRCN: Pair Cancellation).

In the second stage of psychometric network analyses, we split the network structure of the WJ IV COG based on sex and age. The results are shown in Figure 3. The network model tree includes a single split based on age groups. Since sex did not yield significant differences in the network structure, it was not used to create any terminal nodes. We performed structural change tests (Zeileis et al. 2002) to further examine the statistical significance of age and sex in the network model. The results confirmed that age was a statistically significant predictor (structural test statistic = 351.102, *p* < 0.001), whereas sex did not lead to any splits in the network model tree (structural test statistic = 109.295, *p* = 0.1771). This finding suggests that the network structure of the WJ IV COG is homogenous across the samples of female and male participants; however, age leads to significant instabilities in the estimated network parameters. For age, the only split occurred between the group of 6–8-year-olds and the rest of the age groups. This result indicates that the relationship between the subtests of the WJ IV COG, as well as broad cognitive abilities, might be different for young children aged 6 to 8. To further examine the differences between the two network structures split by age, we used the comparetree function in the networktree package. The most significant differences between the two network structures are presented in Table 3.

Table 3 shows that although there is no significant correlation between scores of the Number Series and Picture Recognition in the group of 6–8-year-olds, there is a positive relationship between the same subtests in the remaining age groups. Similarly, there is a stronger relationship between the Concept Formation and Phonological Processing subtests in the group of 6–8-year-olds, compared with the other age groups. Overall, the WJ IV COG network structure is largely age invariant; however, the relationships among the broad cognitive abilities for young school-aged children (age 6 to 8) appear to be different from those among older school-aged children and adolescents (age 9 to 19). 

## 5. Discussion

When the first tests of intelligence emerged in the early 20th century, test developers at that time were mindful of age differences in the performance of students to whom these tests were administered (Saklofske et al. 2015). Over the course of subsequent decades and into the 21st century, researchers continued to report on age-related differences in the development of cognitive abilities (Horn and Cattell 1966b; Horn 2014). Although it may seem that the examination of age-related differences within and across age groups is relatively well-established, there is an ongoing need to continue this work. For example, researchers recognize that tasks used to assess the various aspects of intelligence included in contemporary measures of cognitive abilities consider age differences by incorporating developmentally appropriate content (Wahlstrom et al. 2018). Therefore, with every new or revised measure of intelligence that is published, it is important to establish if, and to what extent, age-based differences exist.

As stated by Taub and McGrew (2004), “establishing an instrument’s factorial invariance provides the empirical foundation to compare an individual’s score across time or to examine the pattern of correlations between variables in differentiated age groups” (p. 71). Extensive evidence of measurement invariance exists for other measures (e.g., Dombrowski et al. 2020; Niileksela et al. 2013), as well as the previous version of the Woodcock-Johnson Tests of Cognitive Abilities (e.g., Benson and Taub 2013; Keith et al. 2008). Although some studies have begun to examine the factor structure of the various CHC abilities represented in the WJ IV (e.g., Dombrowski et al. 2018), evidence for age-based measurement invariance in currently limited.

The WJ IV COG is a comprehensive assessment that measures different aspects of human intelligence based on CHC theory. In this study, we examined the invariance of the relationships among the broad cognitive abilities measured within the WJ IV COG, based on sex and age (the 6- to 19-year age range). Unlike previous studies testing the measurement invariance of intelligence tests based on factor analytic methods, we used psychometric network modeling as an alternative approach to investigate how sex and age affect the network structure of intelligence underlying the WJ IV COG. Using large-scale data from a normative sample of school-aged children and adolescents from the United States population, we first confirmed the hierarchical factorial structure of the WJ IV COG based on CHC theory. We used the latent variable modeling approach that yields a hierarchical structure of broad cognitive abilities (Gc, Gf, Gs, Gwm, Glr, Ga, and Gv) and general intellectual ability (g) within the same model. The results from the hierarchical CFA models indicated that the WJ IV COG is compatible with the hierarchical structure of intelligence with seven broad cognitive abilities (first-order factors), defined from the individual subtests, and general intellectual ability of g (second-order factor), defined from the broad cognitive abilities.

Next, we performed psychometric network modeling to explore the network structure of the broad cognitive abilities measured by the WJ IV COG. Previous studies have discussed the fundamental differences between latent variable modeling and psychometric modeling (e.g., Kan et al. 2019; Schmank et al. 2019; van der Maas et al. 2006, 2017). Unlike latent variable modeling, psychometric network modeling yields an interconnected network structure of cognitive abilities based on the Process Overlap Theory (POT; Kovacs and Conway 2016). To date, several intelligence tests, such as the WAIS-IV and the Brief Test of Adult Cognition by Telephone (Lachman et al. 2014), were analyzed using psychometric network modeling. To the best of our knowledge, this is the first study exploring the network structure underlying the WJ IV COG data. Our findings showed that the subtest scores from the WJ IV COG are positively correlated with each other and that the subtests of Number Series, Letter-Pattern Matching, and Oral Vocabulary play an important role in the network structure of the WJ IV COG. 

Lastly, we used the NMT approach (Jones et al. 2020) to assess whether sex and age could be important factors when interpreting the relationships among the broad cognitive abilities measured by the WJ IV COG subtests. The NMT approach combines psychometric network modeling and model-based recursive partitioning for finding splits in the network structure based on relevant covariates. In this study, we used the NMT approach to recursively split the network structure of the WJ IV COG subtests based on sex and age. This approach enabled us to simultaneously evaluate the impact of sex and age on the invariance of the correlations among the WJ IV COG subtests. Our findings suggested that sex did not lead to any significant differences in the network structure of the WJ IV COG and thus it did not yield any splits. Unlike sex, age was a significant covariate for the network structure of the WJ IV COG. Based on age groups, the network structure was split into two terminal nodes: one for the youngest age group (ages 6 to 8) and another for the remaining age groups (i.e., ages 9 to 19). It is perhaps unsurprising to see the network structure split into two terminal nodes when considering the differences in the developmental slopes associated with those two age ranges. Specifically, the developmental growth that occurs across the seven CHC factor structures, as well as the Gf-Gc composite, is much more significant between the ages of 6 and 8 than it is between the ages of 9 and 19 (p. 136, McGrew et al. 2014). Further analysis of the network model tree, however, shows that the WJ IV COG subtests associated with Fluid Reasoning (Gƒ) and Visual Processing (Gv) are the primary reasons for age-based differences. Given the important role of the Number Series subtest in the network structure (see Figure 2), it is not surprising that the age effect for this subtest led to significant differences in the model. Previous studies showed differential age effects related to the Number Series subtest (e.g., Cormier et al. 2017). In addition to Number Series, this study showed that Concept Formation (Gf) and Picture Recognition (Gv) also appear to be impacted by age.

### Limitations and Future Research

Our study had a few limitations that could be addressed in future research. First, the analyses performed in the current study were based on the United States normative data of the WJ IV COG. Future studies should investigate the impact of sex and age on the network structure of the WJ IV COG subtests across different cultures. Additionally, the cross-sectional nature of the WJ IV COG data precludes the analysis of network trends across age groups over time. Therefore, future studies involving longitudinal data collection with the WJ IV COG are needed to better understand the trends across age groups. The second limitation, as with all studies using psychometric network modeling, is that our analyses followed a data-driven, exploratory approach, instead of a confirmatory approach based on latent variable modeling. That is, we did not attempt to substantiate multi-group comparisons of the broad cognitive abilities and general intellectual ability measured by the WJ IV COG. Therefore, our analyses should not be interpreted as formal tests for measurement invariance across sex and age groups in the WJ IV COG. Future research should examine the alignment between psychometric network analyses and traditional measurement invariance analyses based on multi-group CFA models. Third, the findings of our study indicated that the Number Series subtest played an important role in the network structure of the WJ IV COG subtests. This finding is not surprising because the Number Series subtest is associated with multiple intelligence factors: fluid reasoning (Gf), general intellectual ability (g), and brief intellectual ability (Schrank et al. 2014). However, the strength of the relationship between Number Series and other subtests appears to change depending on age. Therefore, future research is needed to further elucidate why the associations between Number Series and other subtests vary with age. Lastly, this study only focused on sex and age differences in the network structure of the WJ IV COG. Future studies should include other relevant covariates, such as race, ethnicity, and socio-economic status.

## Figures and Tables

**Table 1:** Demographics of the participants.

Characteristics	*n*	%
Sex		
Male	2075	49.3
Female	2137	50.7
Race		
African American	609	14.5
American Indian	31	0.7
Asian or Pacific Islander	190	4.5
Other	93	2.2
White	3289	78.1
Hispanic Origin		
Hispanic	736	17.5
Non-Hispanic	3746	82.5
Geographic Region		
Northeast	716	17
Midwest	1060	25.2
South	1340	31.8
West	1096	26
Parent’s Education Level		
Less than high school	450	10.7
High school graduate	1387	32.9
More than high school	2375	56.4

**Table 2:** Model fit indices for the hierarchical confirmatory factor models of the WJ IV COG.

Models	χ^2^	*df*	CFI	TLI	RMSEA	SRMR	AIC	BIC
Overall Model	2657.765 *	70	0.945	0.929	0.094	0.031	478,632	478,943
Sex								
Male	1282.733 *	70	0.950	0.935	0.091	0.031	235,279	235,555
Female	1483.616 *	70	0.939	0.921	0.097	0.033	242,944	243,222
Age Groups								
6–8	686.962 *	70	0.907	0.880	0.096	0.043	110,121	110,358
9–11	656.021 *	70	0.888	0.854	0.094	0.050	104,950	105,188
12–14	612.471 *	70	0.882	0.850	0.092	0.047	102,110	102,346
15–19	948.792 *	70	0.887	0.853	0.095	0.050	154,293	154,548

Note: χ^2^ = Model chi-square statistic. *df* = Degrees of freedom. CFI = Comparative fit Index. TLI = Tucker-Lewis index. RMSEA = Root mean square error of approximation. SRMR = Standardized root mean squared residual. AIC = Akaike information criterion. BIC = Bayesian information criterion. * *p* < 0.001.

**Table 3:** Significant differences between the values of the edges of the network structures.

Node 1	Node 2	Group 1 (Age 6 to 8)	Group 2 (Age 9 to 19)	Groups 1–2
NUMSER	PICREC	0.00	0.12	−0.12
CONFRM	PHNPRO	0.14	0.05	0.09
PHNPRO	STYREC	0.00	0.08	0.08
GENINF	ORLVOC	0.53	0.61	−0.08
PICREC	VAL	0.16	0.08	0.08

Note: ORLVOC: Oral Vocabulary. NUMSER: Number Series. PHNPRO: Phonological Processing. STYREC: Story Recall. GENINF: General Information. CONFRM: Concept Formation. VAL: Visualization. PICREC: Picture Recognition.

## Data Availability

Data used in this study were obtained from Riverside Insights (formerly, Riverside Publishing). All data are solely owned and licensed by Riverside Insights and thus cannot be shared by the authors in any form or format. Requests to access the data should be directed to Riverside Insights.
